# High-throughput proteomic profiling of the fish liver following bacterial infection

**DOI:** 10.1186/s12864-018-5092-0

**Published:** 2018-10-01

**Authors:** Dwight R Causey, Moritz A N Pohl, David A Stead, Samuel A M Martin, Christopher J Secombes, Daniel J Macqueen

**Affiliations:** 1 School of Biological Sciences, University of Aberdeen, Aberdeen, UK; 2 Aberdeen Proteomics, University of Aberdeen, The Rowett Institute, Aberdeen, UK

**Keywords:** Label-free proteomics, Hybrid quadrupole-Orbitrap mass spectrometry, Immune system, Rainbow trout, *Aeromonas salmonicida*, Complement system, Complement C3, Gene duplication

## Abstract

**Background:**

High-throughput proteomics was used to determine the role of the fish liver in defense responses to bacterial infection. This was done using a rainbow trout (*Oncorhynchus mykiss*) model following infection with *Aeromonas salmonicida*, the causative agent of furunculosis. The vertebrate liver has multifaceted functions in innate immunity, metabolism, and growth; we hypothesize this tissue serves a dual role in supporting host defense in parallel to metabolic adjustments that promote effective immune function. While past studies have reported mRNA responses to *A. salmonicida* in salmonids, the impact of bacterial infection on the liver proteome remains uncharacterized in fish.

**Results:**

Rainbow trout were injected with *A. salmonicida* or PBS (control) and liver extracted 48 h later for analysis on a hybrid quadrupole-Orbitrap mass spectrometer. A label-free method was used for protein abundance profiling, which revealed a strong innate immune response along with evidence to support parallel rewiring of metabolic and growth systems. 3076 proteins were initially identified against all proteins (*n* = 71,293 RefSeq proteins) annotated in a single high-quality rainbow trout reference genome, of which 2433 were maintained for analysis post-quality filtering. Among the 2433 proteins, 109 showed significant differential abundance following *A. salmonicida* challenge, including many upregulated complement system and acute phase response proteins, in addition to molecules with putative functions that may support metabolic re-adjustments. We also identified novel expansions in the complement system due to gene and whole genome duplication events in salmonid evolutionary history, including eight C3 proteins showing differential changes in abundance.

**Conclusions:**

This study provides the first high-throughput proteomic examination of the fish liver in response to bacterial challenge, revealing novel markers for the host defense response, and evidence of metabolic remodeling in conjunction with activation of innate immunity.

**Electronic supplementary material:**

The online version of this article (10.1186/s12864-018-5092-0) contains supplementary material, which is available to authorized users.

## Background

Vertebrate immune function requires coordination of a complex set of regulatory processes and signaling pathways. The immediate innate response to pathogenic insult is underpinned by cellular and humoral components that are fairly well characterized in teleost fishes [1, 2], with many known contributing genetic components [3]. For example, the conserved cytokines IL-1β, IL-8 and tumor necrosis factor α (TNFα) each activate nuclear factor kappa-light-chain enhancer of activated B cells (NF-κB) signaling pathways to regulate early inflammatory responses to bacterial infection [4], which are followed by the acute phase response (APR), defined by production of plasma proteins such as complement system components, cerebellin-like proteins, lectins, haptoglobin and ferritin [5, 6].

The innate immune response of teleost fishes occurs primarily in lymphoid organs such as head kidney and spleen, and a variety of mucosal-associated sites (e.g. gills, gut, skin and nostrils) [7, 8], which produce cells and humoral parameters responsible for clearing a pathogen [1]. Monocytes, macrophages, and neutrophils degrade pathogenic particles through phagocytosis, while nonspecific cytotoxic cells help eliminate pathogens by apoptosis [9]. Humoral innate immune components include a wide range of receptors and molecules that are soluble in plasma and other body fluids, consisting of cytokines, APR proteins, antimicrobial peptides and protease inhibitors, among others [1]. Despite its traditional perception as a metabolic, nutrient storage, and detoxification center, the vertebrate liver is also an important immune organ and produces cytokines, chemokines, complement components and APR proteins in response to pathogenic challenge (reviewed in [10]). Resident immune cells contributing to liver immune function include macrophages (Kupffer cells), neutrophils, B lymphocytes, T lymphocytes and NK cells [11]. The liver’s dual role in immune function and metabolism makes it an interesting candidate for linking host defense with metabolic readjustments upon pathogen challenge.

Significant energy reserves are required to maintain immune function and to activate defense responses, for example the production of high titres of pro-inflammatory cytokines and APR proteins [5, 12, 13]. Energetic reserves allocated towards an immune response cannot be invested in other physiological systems, including anabolic processes linked to growth and reproduction, which may be essential to survival and fitness. Given that such systems also demand significant energetic resources, trade-offs between immune and other functions should have evolved under selection to maximize fitness and survival depending on environmental conditions (e.g. the presence or absence of a pathogen) and life-history strategy [14]. In turn, such trade-offs presumably require ‘cross-talk’ between different physiological systems to facilitate re-mobilization of energy towards an effective immune response upon infection [15]. In teleosts, there is growing evidence for such interrelationships between immune function and key pathways regulating growth [16–18], as well as stress (see [19–21]), thyroid function [22, 23] and other endocrine factors (reviewed by [24]).

Here we hypothesize that the liver plays a dual role in supporting the host defense response during infection, while mediating parallel metabolic adjustments that promote rewiring of energetic resources towards immune function. As a study system we used rainbow trout (*Oncorhynchus mykiss*) exposed to *Aeromonas salmonicida*, the causative agent of furunculosis, a disease with profound negative effects on aquacultured fish including salmonids. Past studies of salmonids have characterized responses to bacterial pathogens using transcriptomic analyses of different tissues, including liver [25–27], spleen [27, 28], head kidney [25, 27] and gill [25]. Additional research has documented the effects of bacterial pathogens in various fish immune tissues using proteomics [28–30], though these studies are yet to include liver. We have employed high-throughput proteomics to discover a set of proteins showing differential abundance in rainbow trout liver following *A. salmonicida* infection, revealing many known and novel markers for host defense, along with candidate proteins that may support cross-talk between immune function and metabolism.

## Results

### Experimental design and validation of immune response

**Fig. 1:**
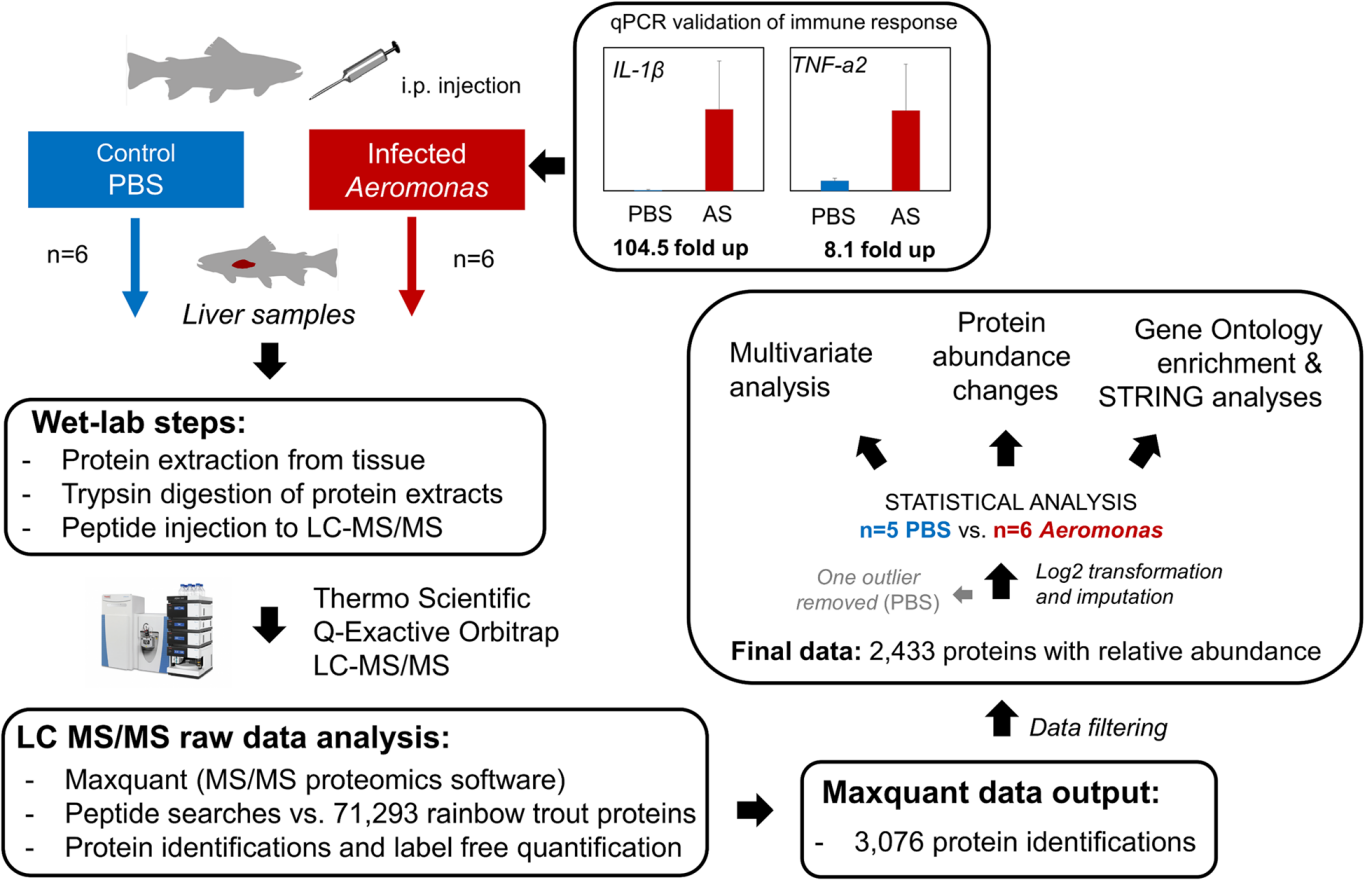
Summary of study experimental design. For full details see the main text

The experimental design of our study is summarized in Fig. 1. We performed a high-throughput proteomic analysis of rainbow trout liver comparing controls (48 h after PBS-injection) to bacterial-challenged (48 h post-infection of *Aeromonas salmonicida;* ‘AS’) samples (Fig. 1). While we did not confirm liver pathology in the AS-challenged trout, past studies in other fish species have provided evidence for liver pathology post-infection with another *Aeromonas* species (*A. hydrophila)* at similar timescales (e.g. for catfish: [31]; for Golden Mahseer: [32]). In this study, to validate that a systematic immune response occurred to AS challenge, we used quantitative PCR gene expression profiling to demonstrate a strong transcriptional up-regulation of the pro-inflammatory cytokines *IL-1β* and *TNF-α2* in head kidney (i.e. primary immune tissue) sampled from the same animals (see Fig. 1). Specifically, *IL-1β* and *TNF-α2* increased by approx. 105- and 8.1-fold, respectively, in the AS samples compared to controls (respective one-way ANOVA tests: *P* < 0.0001 and *P* < 0.001). The strong response of these immune markers in head kidney is consistent with a systematic immune response to AS infection in the animals used for proteomics. In addition, we observed a markedly enlarged spleen in the AS treated animals only (not shown), which is a clinical sign of bacterial infection (e.g. [33]), and further indicates a successful infection challenge.

Our choice to sample animals 48 h post-AS challenge is supported by past work done by authors on this paper, which reported the temporal dynamics of liver and head kidney responses to AS challenge in rainbow trout using a similar infection model, including expression responses for several immune genes [25]. That study revealed a robust induction of immune genes at 6 h, 12 h, 24 h and 48 h, with some variation across time-points [25]. By selecting a single sampling time point, we acknowledge that our study fails to capture the full temporal dynamic of bacterial infection, but nonetheless, taken with our gene expression profiling data at 48 h (Fig. 1) we can be confident that a mature immune response was underway, providing a strong basis for proteomic exploration.

### Global liver proteome response to bacterial challenge

**Fig. 2:**
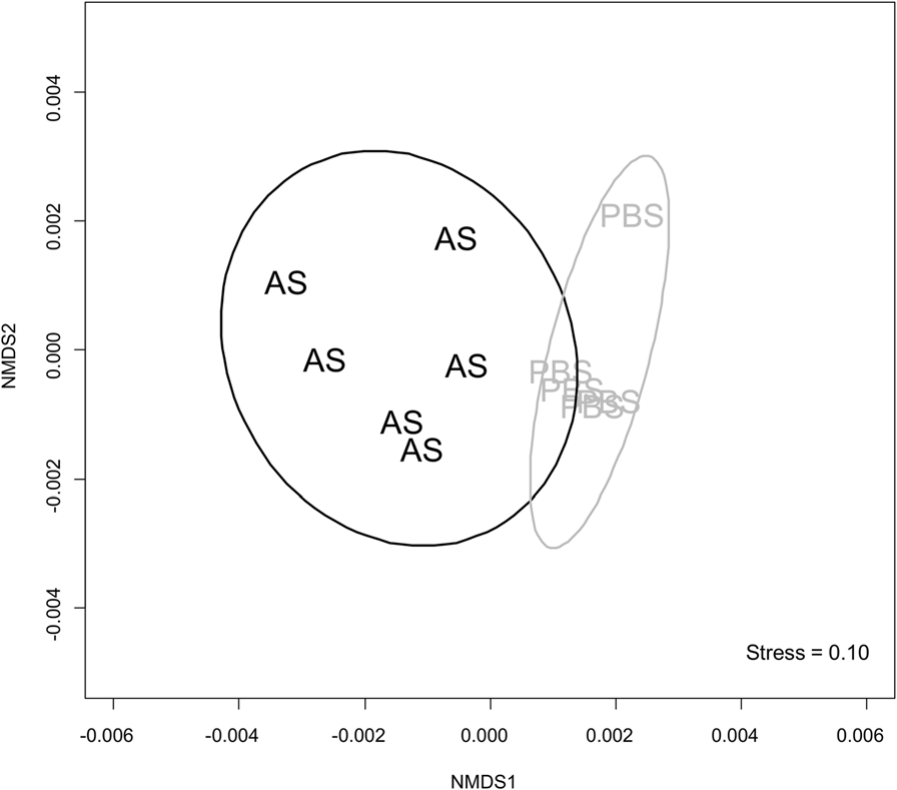
Nonmetric multidimensional scaling showing separation of AS vs. PBS (control) injected rainbow trout. Each label represents an individual fish and their entire quantified liver proteome. The proximity of labels indicates the relatedness of proteomic profiles among individuals. Ellipses represent 95% confidence intervals

Our high-throughput proteomics analysis of rainbow trout liver led to the initial identification of 3076 proteins (Additional File 1: Table S1), 2433 of which were maintained for statistical analysis after quality control steps (Methods; data in Additional file 1: Table S2). Nonmetric multidimensional scaling was used to view all samples in the same multivariate space, revealing a clear separation of individuals from the AS and control groups (Fig. 2). Separation of AS and control samples at the proteomic level was confirmed using a PERMANOVA (9999 permutations, *pseudo*-F_1,9_ = 3.07, *P* = 0.002). Overall, these multivariate analyses demonstrate a major proteomic remodeling of rainbow trout liver following AS challenge.

### Proteins responsive to bacterial challenge

**Fig. 3:**
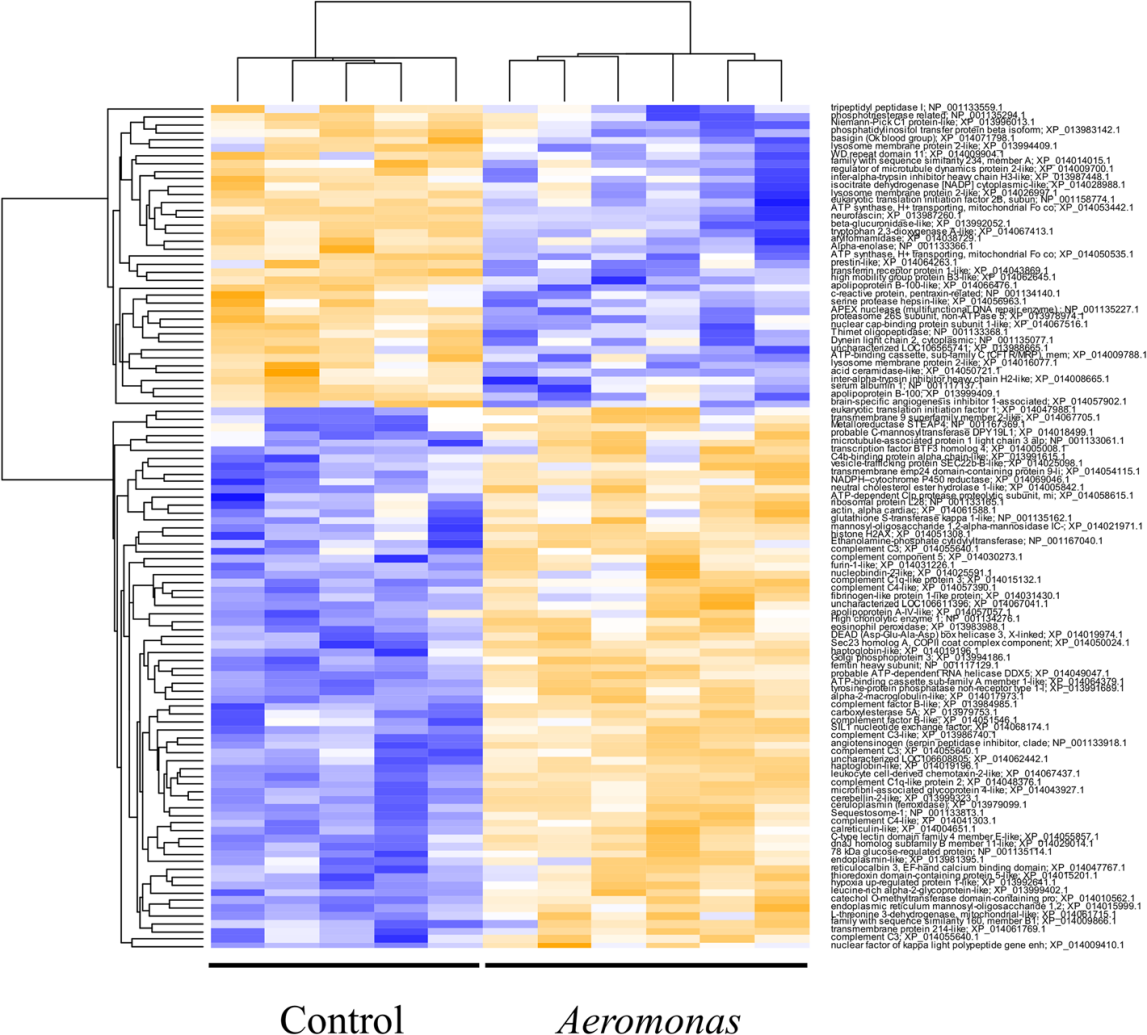
Hierarchical clustering of significantly differentially abundant proteins between AS (*n* = 6) and PBS/control (*n* = 5) in rainbow trout liver. Rows are normalized Z-scores of imputed LFQ values

Using a general linear model separately for all proteins in our dataset*,* 69 and 109 proteins showed differential abundance between the AS and control groups at two significance cut-offs correcting for multiple comparisons (FDR-adjusted *P* < 0.05 and *P <* 0.1, respectively) (Full data in Additional file 1: Table S3). Given the large number of proteins in our dataset, the FDR adjustment is likely very stringent, so we tolerated the higher type-I error rate (i.e. FDR-adjusted *P <* 0.1) to allow inclusion of a greater number of proteins in downstream analyses (Table 1), particularly when considering that known immune proteins were included in the list falling between *P* = 0.05 and 0.1. Hierarchical clustering revealed a clear separation of AS and PBS samples, with 70 upregulated and 39 downregulated proteins (Fig. 3). The upregulated proteins includes many with putative roles in innate immunity (e.g. complement proteins, leukocyte cell-derived chemotaxin-2-like, C-type lectin, alpha2-macroglobulin, nuclear factor of kappa light polypeptide gene enhancer in B-cells 2 [NF-κB]), innate immune accessory proteins (e.g. sequestosome, haptoglobin, eosinophil peroxidase, ferritin heavy subunit, furin-1), translation and transcription factors (e.g. eukaryotic translation initiation factor 1, transcription factor BTF3 homolog 4), transport proteins (e.g. Golgi phosphoprotein 3, phosphatidylinositol transfer protein beta isoform-like), hydrolases (e.g. tyrosine-protein phosphatase non-receptor type 1-like, phosphotriesterase related), proteins involved in the stress response (e.g. 78 kDa glucose-regulated protein, SIL1 nucleotide exchange factor, dnaJ homolog subfamily B member 11-like, endoplasmin-like), and the metalloreductase STEAP4 (STAMP2) (Table 1, Additional file 1: Table S3).

**Table 1:** Proteins showing significantly differential abundance between AS-challenged and control fish, given in descending order of Log2 fold-change

NCBI accession	Protein product	Fold-change (Log2)
XP_021480534	Complement c1q-like protein 2	3.917
XP_021446773	Eosinophil peroxidase	3.915
XP_021438648	Leukocyte cell-derived chemotaxin-2-like	2.837
XP_021466891	Cerebellin-2-like	2.756
XP_021441170	High choriolytic enzyme 1	2.565
XP_021417046	Sequestosome-1	2.417
XP_021442122	Ferritin heavy subunit	2.411
XP_021462825	Haptoglobin-like	2.359
XP_021451323	Microfibril-associated glycoprotein 4-like	2.133
XP_021417220	Complement C3	2.054
XP_021439265	SIL1 nucleotide exchange factor	1.992
XP_021441697	Haptoglobin-like	1.979
XP_021417240	Complement C3	1.917
XP_021469499	Catechol O-methyltransferase domain-containing protein 1-like	1.698
XP_021475009	ATP-binding cassette sub-family A member 1-like	1.446
XP_021436350	Uncharacterized LOC106608805	1.350
XP_021474674	Histone H2AX	1.291
XP_021431988	Angiotensinogen (serpin peptidase inhibitor, clade A, member 8)	1.273
XP_021449457	Probable C-mannosyltransferase DPY19L1	1.263
XP_021417877	C-type lectin domain family 4 member E-like	1.199
XP_021454428	Nucleobindin-2-like	1.183
XP_021458892	Ribosomal protein L28	1.148
XP_021475868	Complement c1q-like protein 3	1.133
XP_021413091	Metalloreductase STEAP4	1.069
XP_021416974	Mannosyl-oligosaccharide 1,2-alpha-mannosidase IC-like	1.061
XP_021454217	L-threonine 3-dehydrogenase, mitochondrial-like	0.996
XP_021474489	Complement factor B-like	0.979
XP_021442709	Alpha-2-macroglobulin-like	0.966
XP_021423951	Uncharacterized LOC106611396	0.962
XP_021423876	Complement C3-like	0.935
XP_021467115	Actin, alpha cardiac	0.923
XP_021445885	Complement factor B-like	0.882
XP_021480047	Apolipoprotein A-IV-like	0.858
XP_021464723	Nuclear factor of kappa light polypeptide gene enhancer in B-cells 2, p49/p100	0.853
XP_021467190	Leucine-rich alpha-2-glycoprotein-like	0.811
XP_021481050	Probable ATP-dependent RNA helicase DDX5	0.772
XP_021460419	Ceruloplasmin (ferroxidase)	0.757
XP_021479504	Hypoxia up-regulated protein 1-like	0.715
XP_021430114	Fibrinogen-like protein 1-like protein	0.714
XP_021417252	Complement C3	0.680
XP_021425166	Carboxylesterase 5A	0.662
XP_021481346	ATP-dependent Clp protease proteolytic subunit, mitochondrial-like	0.662
XP_021451946	Complement C4-like	0.650
XP_021479838	Transcription factor BTF3 homolog 4	0.637
XP_021462359	Complement component 5	0.625
XP_021442137	Complement C4-like	0.591
XP_021453075	Dnaj homolog subfamily B member 11-like	0.581
XP_021480381	Eukaryotic translation initiation factor 1	0.575
XP_021475794	Thioredoxin domain-containing protein 5-like	0.569
XP_021464769	Microtubule-associated protein 1 light chain 3 alpha	0.563
XP_021461669	Family with sequence similarity 160, member B1	0.558
XP_021428745	Neutral cholesterol ester hydrolase 1-like	0.537
XP_021454752	Vesicle-trafficking protein SEC22b-B-like	0.513
XP_021446601	78 kda glucose-regulated protein	0.511
XP_021476867	Endoplasmic reticulum mannosyl-oligosaccharide 1,2-alpha-mannosidase-like	0.481
XP_021481745	Reticulocalbin 3, EF-hand calcium binding domain	0.478
XP_021478379	Golgi phosphoprotein 3	0.477
XP_021441988	Furin-1-like	0.454
XP_021473449	Glutathione S-transferase kappa 1-like	0.437
XP_021460947	Endoplasmin-like	0.418
XP_021438958	Transmembrane emp24 domain-containing protein 9-like	0.403
XP_021421845	Tyrosine-protein phosphatase non-receptor type 1-like	0.398
XP_021455883	Calreticulin-like	0.381
XP_021434540	DEAD (Asp-Glu-Ala-Asp) box helicase 3, X-linked	0.378
XP_021456822	Transmembrane protein 214-like	0.376
XP_021420055	C4b-binding protein alpha chain-like	0.365
XP_021439514	Transmembrane 9 superfamily member 2-like	0.354
XP_021429629	Sec23 homolog A, COPII coat complex component	0.323
XP_021412244	Ethanolamine-phosphate cytidylyltransferase	0.315
XP_021437539	NADPH--cytochrome P450 reductase	0.306
XP_021451429	ATP synthase, H+ transporting, mitochondrial Fo complex, subunit F2	−0.257
XP_021472646	Alpha-enolase	−0.286
XP_021463425	Regulator of microtubule dynamics protein 2-like	−0.298
XP_021414705	Brain-specific angiogenesis inhibitor 1-associated protein 2-like	−0.331
XP_021418673	Thimet oligopeptidase	−0.333
XP_021458913	Proteasome 26S subunit, non-atpase 5	−0.362
XP_021419800	APEX nuclease (multifunctional DNA repair enzyme) 1	−0.366
XP_021469331	Niemann-Pick C1 protein-like	−0.366
XP_021472978	Dynein light chain 2, cytoplasmic	−0.378
XP_021419888	Inter-alpha-trypsin inhibitor heavy chain H2-like	−0.378
XP_021430578	ATP synthase, H+ transporting, mitochondrial Fo complex, subunit s (factor B)	−0.379
XP_021439703	Nuclear cap-binding protein subunit 1-like	−0.382
XP_021424380	Inter-alpha-trypsin inhibitor heavy chain H3-like	−0.403
XP_021425476	Uncharacterized LOC106565741	−0.405
XP_021459433	Basigin (Ok blood group)	−0.425
XP_021439789	Tryptophan 2,3-dioxygenase A-like	−0.426
XP_021474939	Acid ceramidase-like	−0.438
XP_021461466	WD repeat domain 11	−0.439
XP_021421009	C-reactive protein, pentraxin-related	−0.480
XP_021458562	Phosphatidylinositol transfer protein beta isoform-like	−0.482
XP_021439959	Tripeptidyl peptidase I	−0.482
XP_021433346	High mobility group protein B3-like	−0.495
XP_021479945	Beta-glucuronidase-like	−0.522
XP_021478259	Lysosome membrane protein 2-like	−0.525
XP_021412279	Arylformamidase	−0.526
XP_021444801	Phosphotriesterase related	−0.531
XP_021461213	Lysosome membrane protein 2-like	−0.573
XP_021478257	Lysosome membrane protein 2-like	−0.582
XP_021481136	Serine protease hepsin-like	−0.584
XP_021453070	Isocitrate dehydrogenase [NADP] cytoplasmic-like	−0.593
XP_021469787	Eukaryotic translation initiation factor 2B, subunit 5 epsilon, 82kda	−0.594
XP_021470341	Serum albumin 1	−0.649
XP_021460202	ATP-binding cassette, sub-family C (CFTR/MRP), member 2	−0.665
XP_021420795	Family with sequence similarity 234, member A	−0.688
XP_021466049	Neurofascin	−0.718
XP_021440760	Apolipoprotein B-100-like	−0.738
XP_021419747	Prestin-like	−0.810
XP_021445727	Transferrin receptor protein 1-like	−0.881
XP_021467201	Apolipoprotein B-100	−0.883

**Fig. 4:**
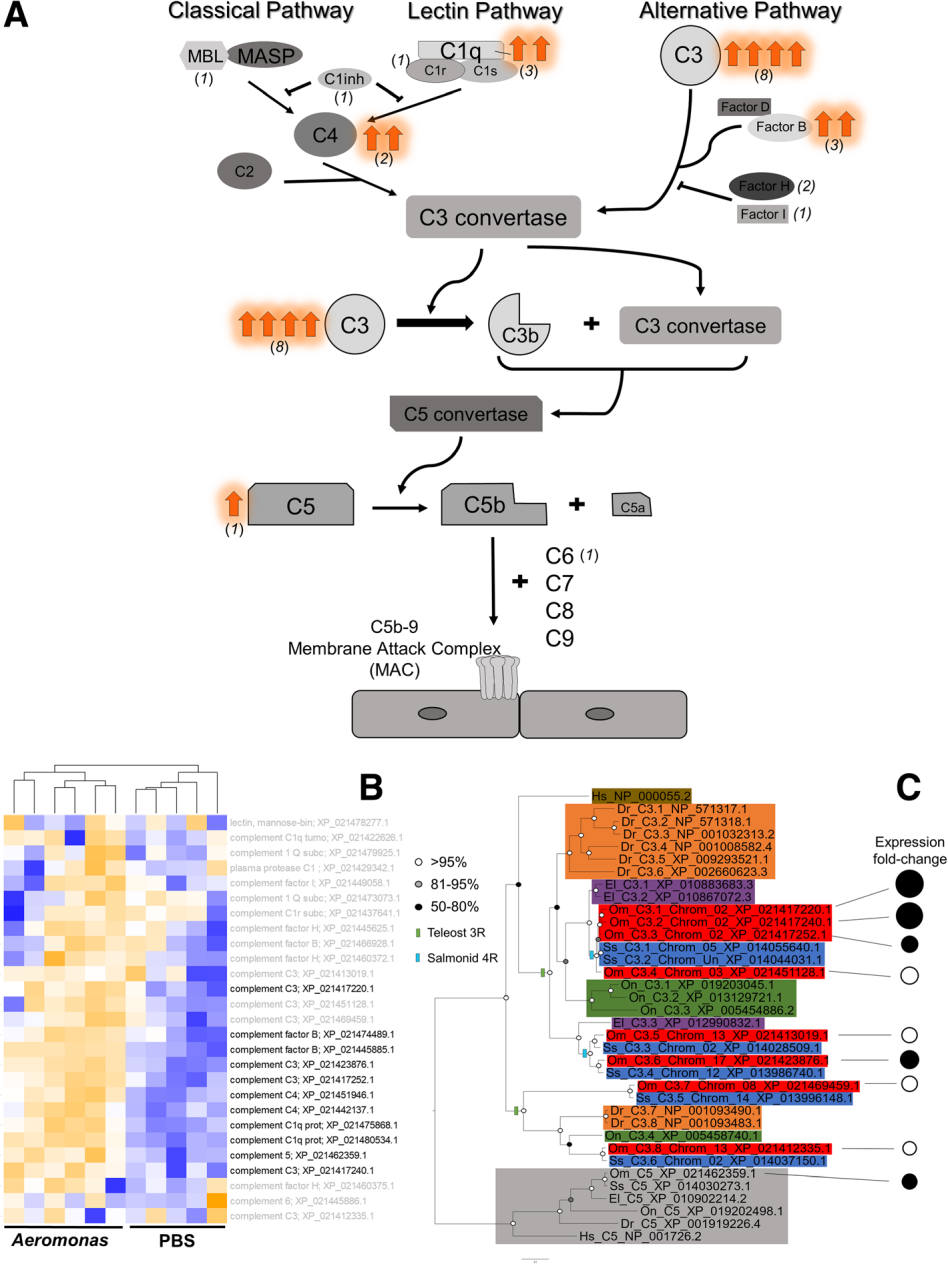
Response of the complement system in liver of AS-challenged rainbow trout. **a** Complement system pathway annotated with protein components quantified in our study. The numbers in parentheses highlight how many unique proteins were identified in our dataset for each complement component. The number of significantly upregulated complement proteins for each system component is shown by orange arrows. **b** Clustering of all identified complement system proteins; titles in bold show significantly differential abundance for AS (*n* = 6) vs. PBS (*n* = 5). **c** Maximum likelihood phylogenetic analysis of C3 proteins from: Human *Homo sapiens* (“Hs”), zebrafish *Danio rerio* (“Dr”), tilapia *Oreochromis niloticus* (“On”), northern pike *Esox lucius* (“El”, a sister lineage to salmonids that did not undergo the salmonid-specific whole genome duplication [53]), Atlantic salmon *Salmo salar* (“Ss”), and rainbow trout *Oncorhynchus mykiss* (“Om”). The tree is annotated to show potential teleost genome duplication events in the teleost (“3R”) and salmonid ancestor (“4R”). Branch support values are shown as circles on each node. Vertebrate C5 proteins provided a validated outgroup to vertebrate C3 proteins [55]. Abundance fold-change values for proteins identified in this study are displayed using bubble plots, with closed circles depicting significantly upregulated proteins

Notably, 11 of the proteins upregulated in AS were from the complement system, representing ~ 10% of all differentially abundant proteins and > 40% of all complement system proteins identified in our dataset (Fig. 4). Identified complement proteins were mapped onto a characterized pathway to highlight the potential impacts of increased abundance on bactericidal activity (Fig. 4a). A phylogenetic analysis was performed with eight unique complement C3 proteins identified in our analysis, which revealed novel teleost and salmonid specific paralogues showing distinct levels of upregulation (Fig. 4c). Interestingly, three significantly upregulated C3 proteins in rainbow trout liver are encoded by genes on chromosome Om02 that were evidently expanded by tandem duplication after divergence between *Oncorhynchus* and *Salmo* (Fig. 4c). In addition, our analysis distinguished distinct complement C3 proteins encoded by rainbow trout gene duplicates retained on different chromosomes, which according to our phylogenetic analysis, are orthologous to Atlantic salmon (*Salmo salar*) genes located within genomic regions retained from the salmonid-specific whole genome duplication event [34] (Fig. 4c).

The more limited set of 39 proteins downregulated by AS included proteins regulating translation (e.g. eukaryotic translation initiation 2B), C-reactive protein, transferrin receptor, beta-glucuronidase-like, lysosome membrane proteins, and apolipoprotein B-100 (Table 1).

### Protein-protein interaction analysis

**Fig. 5:**
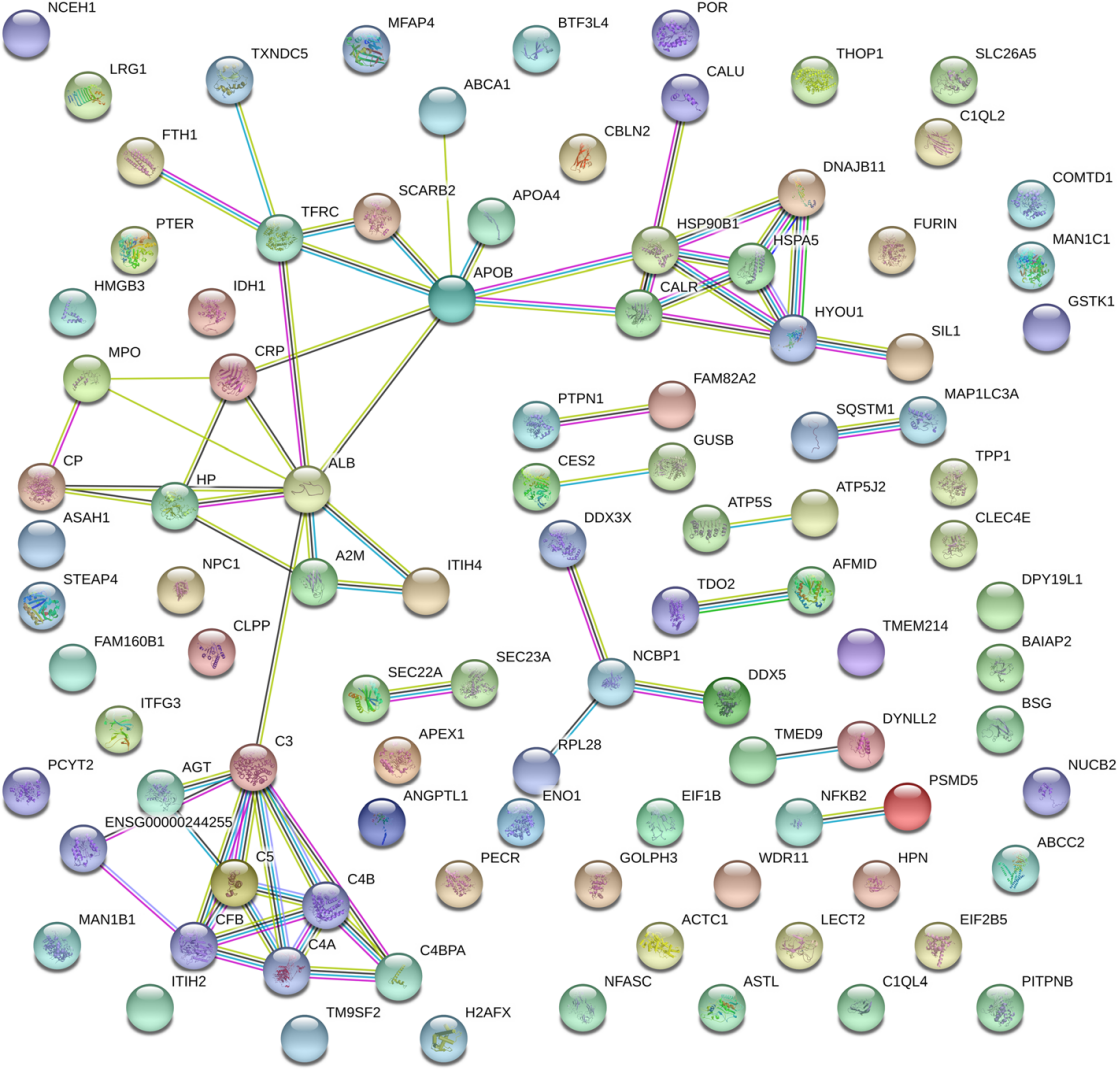
STRING protein-protein interaction network. 109 proteins showing significantly differential abundance between controls and AS were included in the analysis, but only 99 are included in this visual network. Proteins are represented as nodes while interactions appear as edges. The quantity of edges relates to the strength of the interaction relationship

A putative protein-protein interaction (PPI) network was analyzed and a visual representation created using STRING (Fig. 5). This analysis revealed a very significant enrichment (*P* = 1.35e-11) of PPIs among the 109 proteins showing significantly differential abundance between control and AS. The majority of the PPIs (edges) were centered on four main node (protein) clusters, representing the complement (e.g. C3, C4A, C4B, C5), and molecular chaperone systems (e.g. HSP90B1, HSPA5, calreticulin [CALR], DNAJB11), as well as a cluster of APR proteins (e.g. albumin [ALB], haptoglobin [HP], alpha-2-macroglobulin [A2M] and ceruloplasmin [CP]) connected to the molecular chaperone cluster through a smaller cluster of proteins comprised of apolipoprotein B (APOB), transferrin receptor (TFRC) and scavenger receptor class B (SCARB2). Abbreviations and protein annotations from STRING are available in Table S7.

### Gene ontology analyses

We tested whether proteins showing differential abundance between control and AS groups showed an enrichment of particular functions using Gene Ontology (GO) analyses. 103 GO Biological Process (BP) terms showed significant enrichment (Additional file 1: Table S4), many linked to the immune system; such as *complement activation*, *alternative & classical pathway*, *positive regulation of apoptotic cell clearance*, and *acute inflammatory response* (Additional file 1: Table S5). Four complement C3 proteins contributed to the most significant immune-related GOBP enrichments (Additional file 1: Table S5). Other enriched terms included *cholesterol transport*, *negative regulation of metabolic process*, and *response to steroid hormone*. Owing to the complexity of these systems, both upregulated and downregulated proteins contributed to these signals. For example, *cholesterol transport* is predominantly explained by downregulated proteins (6 down vs. 2 up), while *negative regulation of metabolic process* (19 up vs. 13 down) and *response to steroid hormone* (10 up vs. 3 down) are mainly accounted for by upregulated proteins (Additional file 1: Table S5). Several GOBP terms associated with metabolism, including *positive regulation of ERK1 and ERK2 cascade, regulation of triglyceride biosynthetic process*, and *positive regulation of lipid storage* were largely accounted for by the same four complement C3 factors explaining the immune-related GOBP terms (Additional file 1: Table S5).

GO slim analysis revealed 6 significantly enriched terms (Table 2), including *immune system process*, *homeostatic process*, *response to stress*, and *vesicle-mediated transport* (Table 2). A large proportion of proteins are recurrent across terms, contributing ~ 56% of the total explanatory proteins for all 6 significant GO slim terms. Additionally, the GO slim analysis was predominantly driven by upregulated proteins, with ~ 69% of unique contributing proteins showing increased abundance across the 6 significant terms, which predominantly matches with individual GO slim terms (Additional file 1: Table S6).

**Table 2:** Enriched GO slim terms for biological processes for AS-challenged fish

GO slim BP annotation	*P*-value	Odds Ratio ^a^	Count ^b^	Expected ^c^	Size ^d^
Immune system process (GO:0002376)	0.002	1.96	38	24.59	540
Protein maturation (GO:0051604)	0.006	2.88	10	4.01	88
Homeostatic process (GO:0042592)	0.022	1.73	23	15.16	333
Response to stress (GO:0006950)	0.038	1.47	53	43.95	965
Vesicle-mediated transport (GO:0016192)	0.047	1.55	25	18.08	397
Extracellular matrix organization (GO:0030198)	0.048	2.28	7	3.37	74

^a^ *A higher odds ratio indicates enrichment for AS-challenged fish*

^b^ *Number of significantly differentially abundant proteins associated within the GO slim term*

^c^ *Anticipated number of GO terms based on the given data set*

^d^ *Total number of proteins associated with the GO slim term*

## Discussion

High-throughput proteomics is gaining rapid traction in teleost physiology [35–40], underpinned by the rapid progression in generation of new genomics resources, for example reference genomes for multiple salmonid lineages with high-quality proteome predictions (e.g. [41]). Our current study highlights the power of such approaches, revealing many proteins with altered abundances in rainbow trout liver following bacterial infection, including molecules supporting innate immune defense (notably, the APR) and candidate proteins that potentially facilitate energetic re-allocation towards immune function. These findings have implications for our understanding of salmonid health in aquaculture, where bacterial infections such as *A. salmonicida* cause major issues; the proteins we identified may serve as valuable markers both for infection and vaccination responses, and also as candidate immunostimulants that could be explored with the aim of boosting vaccination responses or disease resistance.

Previous proteomics studies of fish responses to bacterial pathogens have not considered liver, instead focusing on spleen, kidney, and intestinal mucosa [28–30]. A recent high-throughput analysis of Atlantic salmon reported remodeling of the liver proteome in response to elevated temperature [42], identifying a number of proteins in common with our study, however the major difference in treatment compared to our immune-focused study limits a useful biological interpretation of parallel changes in protein abundance. Nonetheless, it is interesting that leukocyte cell-derived chemotaxin 2, which was highly upregulated in our study following AS challenge, was strongly downregulated in salmon liver following thermal stress. Our findings are consistent with previous mRNA expression profiling of teleost tissues exposed to bacterial pathogens [25–27, 43–46]. For example, many APR proteins that increased in our study were reported as upregulated in transcriptomic studies of liver responses to *A. salmonicida*, including complement proteins, haptoglobin, ferritin and cerebellin-like protein [25, 27, 43]. Additionally, several non-immune proteins, including 78 kDa glucose-regulated protein precursor (GRP 78), dnaJ homolog subfamily B member 11-like, serine protease, and endoplasmin all showed congruent responses across studies [25, 27, 43]. However, there was not always a direct relationship between liver transcript responses and protein abundance changes across studies. For example, glutathione S-transferase kappa 1 showed upregulation here, but was previously reported to decrease at the mRNA level [25]. It is important to note differences that confound comparisons with past work, including starvation [43] and a distinct experimental infection route [27], while Martin et al. [25] used an attenuated *A. salmonicida* strain known to promote the development of immunological memory [47].

A notable study finding was the strong upregulation of a genetically expanded salmonid complement system following AS challenge. The complement system is activated through the classical, alternative and lectin pathways [48, 49], leading to bactericidal actions through pathogen opsonization, phagocytic activity, and eventual lysis [50–52]. Our analysis distinguished eight C3 proteins (four that were significantly upregulated) encoded by distinct genes, which is a significant expansion on past studies that have reported three distinct C3 proteins in rainbow trout [53, 54]. Our phylogenetic analysis indicated origins for the novel C3 proteins via a history of gene duplication events involving both small-scale and whole genome duplication (WGD) mechanisms, consistent with a past study focused on zebrafish [55] that revealed an ancient teleost-specific C3 member that was identified in our analysis. WGD events are well established in salmonid evolution, including an ancestral autotetraploidization that occurred 88–103 Mya (e.g. [56]). We also observed a > 15-fold upregulation of complement component 1q (C1q), with moderate increases in protein abundance for additional complement components beyond C3, including C4, C5 and factor B. C1q plays a key role in the classical pathway, linking the innate and adaptive systems by binding immunoglobulin molecules to pathogens [57]. Until recently, complement proteins were thought to serve solely immune functions, but emerging evidence suggests metabolic functions, mainly though insulin-like roles and by facilitating triglyceride metabolism [58, 59]. Such roles may contribute to metabolic changes during bacterial infection in rainbow trout, but further work is needed to test this idea. C-reactive protein (CRP) is another innate immune factor that activates complement pathways [60, 61], assists in clearing apoptotic cells [62], and increases liver Kupffer cell phagocytotic activity [63]. Interestingly, CRP showed downregulation in this study, but this nonetheless agrees with past studies of fish liver exposed to *A. salmonicida* [13, 64, 65]. However, increased CRP was detected in serum of rainbow trout exposed to anti-ectoparasitic chemicals [66] and in common carp (*Cyprinus carpio*) challenged with *A. hydrophila*, but not with *Escherichia coli* lipopolysaccharide [67]. These results suggest either a tissue-specific immune role for CRP or yet to be identified liver functions unrelated to immunity. Additionally, the STRING analysis implicated the complement system as being a tightly connected PPI network. But we caution against over-interpretation of STRING results using salmonids, due to genetic expansions in many protein families caused by ancestral WGD events.

We identified other immune proteins, upregulated by AS treatment, which extend far beyond the complement system. For example, leukocyte cell-derived chemotaxin-2-like, which was > 7-fold increased, contributes to adaptive immunity through its chemotactic properties for neutrophils [68]. C-type lectin domain family 4 member E-like(macrophage-inducible C-type lectin), which increased > 2-fold, is an innate immune receptor that induces expression of proinflammatory cytokines [69, 70]. The gene encoding this protein has shown contradictory expression patterns in past studies of Atlantic salmon liver, being either upregulated [71] or unchanged [46] during AS infection. It is notable that NF-κB (p100/p52 subunit) was upregulated by ~ 2-fold following AS treatment. This transcription factor plays a key role in coordinating pathways that drive immune responses [72] and is rapidly activated by pathogens, stress signals, and pro-inflammatory cytokines, leading to the production of cytokines, chemokines, antimicrobial peptides, stress-response proteins, and anti-apoptotic proteins [72].

Several APR proteins showed altered abundance following AS treatment. The metalloreductase six-transmembrane epithelial antigen of prostate 4 (STEAP4), also known as six-transmembrane protein of prostate 2 (STAMP2), was > 2-fold upregulated, and provides a key role in cellular iron and copper homeostasis, crucial to innate immune function [73]. STAMP2 has been suggested to play an important role in metabolic homeostasis by linking inflammation and nutrient signaling [74, 75]. Ferritin heavy subunit, which binds and sequesters blood plasma iron, was increased > 5-fold in AS, and was previously shown to be upregulated by pro-inflammatory cytokines and bacterial infection [76, 77]. Two distinct proteins identified as haptoglobin, best known for its role in binding free plasma hemoglobin, were robustly increased by AS, agreeing with previous work [25, 27, 43] and the characterized immune functions of this APR [78]. Transferrin receptor 1, which was downregulated in our study, serves as the cellular entry point for transferrin-bound iron [79], reducing the availability of iron for bacterial pathogens [5]. Taken with the lack of transferrin regulation observed in our study, which contradicts past work reporting up-regulation of transferrin by AS [27], it is possible that the transferrin system responds to bacterial infection with a complex temporal dynamic that was not captured by our study.

Protein tyrosine phosphatases (PTPs) have important roles in cellular signaling. One specific PTP of interest for cross-talk between growth and immunity is tyrosine-protein phosphatase non-receptor type 1-like (PTP1B), which was upregulated by AS. PTP1B inhibits glucose uptake in the mammalian liver by dephosphorylating the insulin receptor [80–82], potentially making energetic reserves available to other physiological systems (e.g. immune response). PTP1B can also regulate transcription through the attenuation of leptin and JAK2 signaling, which subsequently activate STAT3 [80, 82–84]. PTP1B can also increase protein synthesis by activating Src, an integral part of the PI3K/Akt pathway [85–88], but no significant difference was measured for Src in this study (Additional file 1: Table S3). Contrary to such anabolic effects, there is also evidence for decreased protein synthesis through increased PTP1B induced phosphorylation of PERK and eukaryotic initiation-factor 2α (eIF2α) in mouse pancreatic cells [89]. Further research also suggests a role for PTP1B in immune function, including by negatively regulating cytokine signaling through the dephosphorylation of JAK2 [90, 91] and by TNFα, which promotes PTP1B expression in the liver, partly through NF-κB [92]. The involvement of PTP1B in both growth and immune function makes it a potential candidate for mediating cross-talk between both systems.

An interesting protein upregulated by AS in the context of potential metabolic re-adjustments is the molecular chaperone 78 kDa glucose-regulated protein (GRP78/HSPA5/BiP), which controls activation of the unfolded protein response (UPR) in the endoplasmic reticulum and is upregulated in response to cellular stress [93, 94]. This protein has also been assigned immune functions, including in a past proteomic analysis of an Atlantic salmon head kidney cell line stimulated with the cytokine interferon-γ [95]. Two other proteins that increased following AS treatment, dnaJ homolog subfamily B member 11-like and SIL1 nucleotide exchange factor, serve as co-chaperones to GRP78 during an UPR [96, 97]. The proteinase inhibitor alpha-2-macroglobulin, ~ 2-fold upregulated by AS infection, interacts with GRP78 at the cell surface to activate the PI3K/Akt, ERK1, and MAPK pathways leading to cellular proliferation [98]. Also involved in this pathway is Akt, which serves to activate NF-κB, leading to anti-apoptotic signaling and cellular survival [98]. These interactions of GRP78 indicate a possible role in the rewiring of energetic resources away from growth and protein synthesis and, via NF-κB, into accelerated immune function.

A further subset of proteins was identified that may assist remodeling of liver metabolism in response to infection. We observed downregulation of the eukaryotic translation initiation factor 2B, subunit 5 epsilon (EIF2B5), which likely reduces translation generally, but concomitantly may increase the translation of stress response mRNAs [99, 100]. Phosphatidylinositol transfer protein beta isoform-like, also decreased in AS, is an important component of the polyphosphoinositide synthesis machinery, which is required for epidermal growth factor signaling [101, 102]. Golgi phosphoprotein 3 was increased in AS and is essential for a properly functioning Golgi and morphology [103], but also enhances signaling of the mTORC1 and mTORC2 complexes through increased phosphorylation of their respective substrates, S6K and Akt-S473 [104], leading to increased translation. ATP-binding cassette sub-family A member 1-like (ABCA1), increased > 2.5-fold in AS, facilitates the transfer of cholesterol and lipophilic molecules across cellular membranes, but also has a complicated relationship with cytokines, with some (interferon-γ and interleukin-1β) inhibiting expression, and others (interleukin-10 and transforming growth factor-β1) promoting ABCA1 expression [105]. Another upregulated protein was probable ATP-dependent RNA helicase DDX5 (p68 DEAD box RNA helicase) which has an important role in transcription initiation, elongation, and post-transcriptional processes [106]. Lastly, apolipoprotein B is the primary protein for transporting and distributing lipids throughout the body, especially cholesterol used for plasma membrane and steroid hormone biosynthesis [107] and is generally decreased during the APR [108], matching the observed downregulation in liver of AS-challenged rainbow trout and potentially facilitating metabolic changes. Previous work has also indicated important immune functions for teleost apolipoprotein A [109], which is consistent with the upregulation of one apolipoprotein A protein in response to AS in our study.

## Conclusions

This study has revealed a range of proteins induced by bacterial infection in rainbow trout liver, along with proteins that may contribute to accompanying metabolic readjustments. While this study focused on total protein levels, future proteomics work should be aimed at better understanding the signaling changes, many at the level of reversible phosphorylation modifications, driving immune responses and cross-talk between immunity and metabolism during infection.

## Methods

### Fish husbandry and injection protocols

Rainbow trout (*n* = 25, approx. Weight: 100 g) were kept in two separate 250 L freshwater tanks at the University of Aberdeen’s aquarium facilities. Water temperature was maintained at 14 °C, and fish were fed a commercial pellet diet at 2% body weight per day. The fish were maintained under these conditions for seven weeks prior to experimental infection challenge. Ten fish (five per tank) were randomly selected to receive either a bacterial or PBS injection (approx. Weight 200 g). The pathogenic Hooke strain of the Gram-negative bacterium *A. salmonicida* (AS) [110] was used for the challenge. Animals were anaesthetized then injected intraperitoneally (i.p.) with 2 × 10^5^ colony forming units (cfu)/mL AS in PBS (0.5 mL/fish). The same volume (0.5 mL) of PBS was injected i.p. as a control. Sampling occurred 48 h post-infection. The fish were killed using a Schedule 1 method following prior anaesthetization using 2-phenoxyethanol (0.1% v/v) and whole liver and head kidney was immediately sampled, flash frozen in liquid nitrogen and stored at -70 °C until analysis. Samples were taken from ten fish per group (*Aeromonas* vs. PBS), from which *n* = 6 biological replicates per group were randomly selected for proteomics (liver), and gene expression profiling to validate a systematic immune response to AS challenge.

### Gene expression validation of immune responses

To validate a systematic immune response to AS challenge, we performed qPCR analyses on first-strand cDNA synthesized from total RNA of head kidney samples matched to the same fish used in proteomics (*n* = 6 control, *n* = 6 AS). RNA extraction and cDNA synthesis were done as detailed elsewhere [111]. qPCR was performed on a Roche LightCycler® 480 using 2× SYBR® Green I (Invitrogen™) qPCR Master Mix, made with a Immolase DNA Polymerase kit (Bioline), using 10 μL reaction mixtures in 384-well plates (Roche), containing 4 μL diluted cDNA in each reaction and 500 nmol of forward and reverse primers (primer details for *IL-1β*, *TNF-a2* and *EF-1α* published in Hu et al. [112]). Raw data were analyzed using LightCycler® 480 Software 1.5.1 (Roche). The copy number of each gene was quantified using internal references, by serial dilution of equimolar amounts of PCR product from each gene. Relative gene expression values were separately calculated by normalizing copy number values for *IL-1β* and *TNF-a2* against *EF-1α* (i.e. reference gene) values. To test for differences between the AS and control samples, a one-way ANOVA was completed in Minitab 18 (Minitab, Inc). As the model residuals either showed non-normality or unequal variances for both target genes, a Box-Cox transformation was performed, leading to data that conformed to the assumptions of normality and equal variances.

### Sample preparation for proteomics

Sample preparation, liquid chromatography–mass spectrometry (LC-MS), data analysis, and statistical analysis were performed as reported previously [40]. Briefly, liver tissue was thawed on ice, weighed and lysis buffer (0.5 M pH 6.8 Tris-HCl, 0.2 M EDTA, 8 M Urea, 0.5 M DTT, 10% v/v Glycerol, 10% v/v NP40, pH 3–10 ampholytes) added for a final ratio of ~ 2 mg/μL. The tissue was ground within the buffer using a micropestle, followed by sonication (Fischer Scientific, Sonic Dismembrator) on ice. The resulting suspension was centrifuged at 13,000 g for 5 min, before the supernatant was separated and stored at -80 °C until further analysis. The supernatant was thawed on ice, diluted 50% with molecular grade water, followed by protein precipitation using a ReadyPrep 2-D clean up kit (Bio-Rad Laboratories) according to the manufacturer’s instructions. The resulting pellet was dissolved in 100 μL of 3–10 pH Reswell buffer (Urea, Thiourea, CHAPS, DTT, MilliQ water, and IPG buffer). 5 μL of 3X dissociation buffer (0.5 M pH 6.8 Tris-HCl, 25% SDS, 2-mercaptoethanol, glycerol) was combined with 10 μL of the Reswell solution and incubated for 5 min at 100 °C. A small, 3 μL aliquot of this solution was run a short distance into a 10% acrylamide 1-D gel, then stained with colloidal Coomassie Blue G250 (Fisher Scientific). The protein band was excised for an in-gel tryptic (Promega, sequencing grade) digestion (Digilab ProGest robot). The resulting peptide solutions were dried via centrifugal evaporation (Savant SpeedVac Plus) then dissolved in 20 μL 0.1% formic acid and centrifuged for 5 min at 14,000 g prior to LC-MS.

### LC-MS

An UltiMate 3000 RSLCnano (Dionex/Thermo Scientific) coupled to a Q Exactive Plus quadrupole-equipped Orbitrap MS/MS system was used to analyze samples, where 4 μL of the tryptic peptide solution was injected per sample. A loading solvent of water/acetonitrile/formic acid (98:2:0.1) with a flow rate of 10 μL/min was used to concentrate peptides on a μ-precolumn (C18 PepMap; 300 μm i.d. × 5 mm). The μ-precolumn was switched to the analytical flow path after 5 min. Peptides were separated at a flow rate of 0.3 μL/min along a C18 PepMap RSLC column (2 μm i.d. × 50 cm) fitted to an EASY-Spray nano ESI source. Two solvents were used to separate peptides: Solvent A constituted water/formic acid (1000:1) and Solvent B water/acetonitrile/formic acid (200:800:1). An increasing proportion of solvent B was used along a gradient for the separation of peptides: 3–10% from 5 to 25 min: 10–45% from 25 to 185 min; 45–90% from 185 to 190 min; 90% from 190 to 205 min, 90–3% from 205 to 210 min, followed by re-equilibration (3% solvent B, 30 min). A “Top 10” data-dependent acquisition (DDA) method was used, beginning at 5 min into the LC method and lasting for 200 min. The electrospray voltage was 1.9 kV, capillary temperature 270 °C and S-lens RF level 60. The MS scans were performed between 375 and 1750 m/z at resolution 70,000 (*m/z* 200) with an automatic gain control of 3E + 6 and maximum injection time of 50 ms. The 10 most intense ions of charge state 2–5 were sequentially selected (isolation window 1.6 *m/z*), followed by fragmentation in the higher-energy collisional dissociation (HCD) cell at a normalized collision energy of 26%. MS^2^ scans were conducted at resolution 17,500, with an automatic gain control of 5E + 4 and maximum injection time of 100 ms. Additional data-dependent settings included; peptide match preferred, exclude isotopes turned on, and a 40 s dynamic exclusion.

### Data analysis

Raw data files from the Q-Exactive were analyzed using MaxQuant (v1.5.3.30; [113]) with a label-free quantification (LFQ) method [114]. MaxQuant default and recommended settings were predominantly used (after: [115]) excepted that unmodified counterpart peptides were not discarded. Trypsin digestion was selected, with a maximum of two missed cleavages. Variable modifications allowed were oxidation of methionine and protein acetylation at the N-terminus, while carbamidomethylation of cysteine was a fixed modification. Peptide and protein identifications were subject to a 1% false-discovery rate (FDR), with a first search mass tolerance for precursor ions set to 20 ppm and a 4.5 ppm setting for the main search. The ‘Match between runs’ option was used to identify missing spectra across samples. Peptides were matched against a high-quality rainbow trout genome annotation including 71,293 RefSeq proteins (NCBI accession; GCA_002163495). Contaminants, reverse identifications, and identifications only by site were filtered from the MaxQuant ‘proteingroups.txt’ file and only proteins that had LFQ values in five samples were kept for statistical analysis. LFQ values were log2 transformed and missing values imputed using missForest, a random forest based non-parametric method [116]. MaxQuant output (Additional file 1: Table S1) and imputed data (Additional file 1: Table S2) can be accessed within the Supplementary Tables (Additional file 1).

### Statistical analysis

R version 3.3.2 (“Sincere Pumpkin Patch”) interfacing with R-studio v1.0.136 (Rstudio, Boston, MA) was used for statistical modeling and graphics production. A linear model was used to determine differences between AS and control treatments, done in the ‘limma’ package with smoothing of the standard errors using an empirical Bayes approach [117] and an applied false-discovery rate of 0.1 (rationale in Results section). Heatmaps were produced by comparing Z-scores of normalized LFQ values using the ‘gplots’ and ‘seriation’ packages [118, 119]. Hierarchical clustering was achieved by minimizing Hamiltonian path length through optimal leaf ordering [120]. Multivariate analyses were performed on the same filtered, log2 transformed, and imputed LFQ values, using ‘vegan’ [121]. Non-metric multidimensional scaling (nMDS) was used to visualize the data over a PCA to better preserve the distance between data points. Simultaneous changes across the liver proteomes of AS and control samples was determined using a permutational ANOVA (PERMANOVA, 9999 permutations) [122]. The multivariate homogeneity of group dispersion (variance) was assessed and revealed no dispersion effect [123]. One control (PBS) individual grouped with the AS-challenged fish in the nMDS analysis and was taken as an outlier that was removed from the study.

### Gene ontology (GO) analyses

GO enrichment analysis was conducted for significantly differentially abundant proteins identified from the AS vs control comparison. All rainbow trout proteins identified from MaxQuant were used in BLASTp [124] searches against Atlantic salmon RefSeq proteins predicted from the reference ICSASG_v2 genome [34] (NCBI accession: GCA_000233375.4), which are assigned with GO terms [125]. This allowed us to provide all identified rainbow trout proteins with GO terms for use in enrichment analyses (Additional file 1: Table S1). GO biological process (BP) enrichment was determined using the ‘topGO’ package with the ‘weight01’ algorithm and Fisher’s test statistic [126]. GO slim was conducted using the ‘GOstats’ and ‘GSEABase’ packages [127, 128].

### STRING PPI analysis

PPIs were determined using the STRING database (http://string-db.org/) that determines both physical and functional associations between proteins [129]. The subset of 109 proteins that showed significant differential abundance due to AS treatment were entered into the STRING database. The *Homo sapiens* orthologues were determined within the STRING database from each *O. mykiss* amino acid sequence. Default settings were used, with the interaction score set to “high confidence (0.700)”. Each node represents a protein while the edges indicate the strength of the relationship between proteins (i.e. more edges give higher confidence).

### Phylogenetic analyses of C3 proteins

Phylogenetic analysis of rainbow trout C3 proteins identified in our dataset was performed using sequences gathered from a standardized set of vertebrate taxa. We began with the Human C3 protein and used BLASTp [124] against the NCBI non-redundant protein database to extract putative C3 orthologues from a range of teleost taxa. Previous studies identified three rainbow trout C3 proteins [53, 54] and eight zebrafish (*Danio rerio*) C3 proteins [55] that provided key reference points for the analysis. As done previously [55], vertebrate C5 proteins were gathered using the same approach as an outgroup for phylogenetic analyses. The collected protein sequences (*n* = 36) were aligned using Mafft V7 [130] with default settings before alignment quality filtering was completed using the Guidance2 algorithm [131], leading to a high-confidence 1243 amino acid sequence alignment (provided as Additional File 2: Supplementary Dataset 1). The maximum likelihood approach IQ-tree [132] and server [133] was used to determine the best-fitting amino acid substitution model (WAG+F + I + G4) and build a consensus tree employing the same model, along with 1000 ultrafast bootstrap pseudoreplicates [134] to gain branch support values. The consensus tree was visualized and rendered using FigTree V1.4.3 (http://tree.bio.ed.ac.uk/software/figtree/).

## Additional files

Additional File 1: **Table S1.** Results from BLASTp of *Oncorhynchus mykiss* protein sequences against *Salmo salar* and MaxQuant output after filtering contaminants, reverse sequences, proteins identified by site, and proteins with only 1 peptide for identification (3076 proteins). **Table S2.** Imputed protein abundance data after implementing all filtering criteria (2433 proteins). **Table S3.** Full statistical results from the linear model for each of the 2433 proteins included in the analysis. **Table S4.** Full results for GO Biological Process (GOBP) enrichment analysis. **Table S5.** Significantly different proteins that contribute to each significant GOBP term. **Table S6.** Significantly different proteins that contribute to each significant GO slim term. **Table S7.** Abbreviations and proteins annotations from STRING. (XLSX 3388 kb)

Additional File 2: **Supplementary Dataset 1.** Sequence alignment used for phylogenetic analysis of C3 complement sequences. (FASTA 47 kb)

## Abbreviations

ABCA1: ATP-binding cassette sub-family A member 1-like
APR: Acute phase response
AS: *Aeromonas salmonicida*-injected
BP: Biological Process
C1q: Complement component 1q
C3–5: Complement component 3, 4, or 5
cfu: Colony forming units
CRP: C-reactive protein
DDA: Data-dependent acquisition
DDX5: p68 DEAD box RNA helicase
EIF2B5: Eukaryotic translation initiation factor 2B, subunit 5 epsilon
eIF2α: Eukaryotic initiation-factor 2α
Erk: Extracellular signal-regulated kinase
FDR: False-discovery rate
GO: Gene Ontology
GRP78: 78 kDa glucose-regulated protein
HCD: Higher-energy collisional dissociation
i.p.: Interperitoneally
IL: Interleukin
JAK2: Janus kinase 2
LFQ: Label-free quantification
mTORC1 & 2: Mammalian target of rapamycin complex 1 & 2
NF-κB: Nuclear factor kappa-light-chain enhancer of activated B cells
PERK: Protein kinase R (PKR)-like endoplasmic reticulum kinase
PERMANOVA: Permutational ANOVA
PI3K: Phosphatidylinositol-4,5-bisphosphate 3-kinase
PPI: Protein-protein interaction
PTP: Protein tyrosine phosphatase
PTP1B: Tyrosine-protein phosphatase non-receptor type 1-like
S6K: Ribosomal protein S6 kinase
STAMP2: Six-transmembrane protein of prostate 2
STAT3: Signal transducer and activator of transcription 3
STEAP4: Six-transmembrane epithelial antigen of prostate 4
TNFα: Tumor necrosis factor α
UPR: Unfolded protein response
WGD: Whole genome duplication
